# Hello World Deep Learning in Medical Imaging

**DOI:** 10.1007/s10278-018-0079-6

**Published:** 2018-05-03

**Authors:** Paras Lakhani, Daniel L. Gray, Carl R. Pett, Paul Nagy, George Shih

**Affiliations:** 1 Department of Radiology, Sidney Kimmel Jefferson Medical College, Thomas Jefferson University Hospital, Philadelphia, PA 19107 USA; 2 Sidney Kimmel Jefferson Medical College, Philadelphia, PA USA; 3 Department of Radiology, Johns Hopkins University School of Medicine, Baltimore, MD USA; 4 Division of Health Science Informatics, Johns Hopkins University School of Public Health, Baltimore, MD USA; 5 Department of Radiology, Weill Cornell Medical College, New York, NY USA

**Keywords:** Deep learning, Machine learning, Artificial neural networks, Medical imaging

## Abstract

There is recent popularity in applying machine learning to medical imaging, notably deep learning, which has achieved state-of-the-art performance in image analysis and processing. The rapid adoption of deep learning may be attributed to the availability of machine learning frameworks and libraries to simplify their use. In this tutorial, we provide a high-level overview of how to build a deep neural network for medical image classification, and provide code that can help those new to the field begin their informatics projects.

## Introduction

Machine learning has sparked tremendous interest over the past few years, particularly deep learning, a branch of machine learning that employs multi-layered neural networks. Deep learning has done remarkably well in image classification and processing tasks, mainly owing to convolutional neural networks (CNN) [1]. Their use became popularized after Drs. Krizhevsky and Hinton used a deep CNN called AlexNet [2] to win the 2012 ImageNet Large Scale Visual Recognition Challenge (ILSVRC), an international competition for object detection and classification, consisting of 1.2 million everyday color images [3].

The goal of this paper is to provide a high-level introduction into practical machine learning for purposes of medical image classification. A variety of tutorials exist explaining steps to use CNNs, but the medical literature currently lacks a step-by-step source for those practitioners new to the field in need of instructions and code to build and test a network. There are many different libraries and machine learning frameworks available, including Caffe, MXNet, Tensorflow, Theano, Torch and PyTorch, which have facilitated machine learning research and application development [4]. In this tutorial, we chose to use the Tensorflow framework [5] (Tensorflow 1.4, Google LLC, Mountain View, CA) as it is currently the most actively used [6] and the Keras library (Keras v 2.12, https://keras.io/), which a high-level application programming interface that simplifies working with Tensorflow, although one could use other frameworks as well. Currently, Keras also supports Theano, Microsoft Cognitive Toolkit (CNTK), and soon MXNet.

We hope that this tutorial will spark interest and provide a basic starting point for those interested in machine learning in regard to medical imaging. This tutorial assumes basic understanding of CNNs, some Python programming language (Python 3.6, Python Software Foundation, Wilmington DE), and is more of a practical introduction to using the libraries and frameworks. The tutorial will also highlight some important concepts but due to space constraints not cover everything in full detail.

## Hardware Considerations

For larger datasets, you will want a computer that contains a graphics processing unit (GPU) that supports the CUDA® Deep Neural Network library (cuDNN) designed for Nvidia GPUs (Nvidia Corp., Santa Clara, CA). This will tremendously speed up training (up to 75 times faster than a CPU) depending on the model of the GPU [7]. However, for smaller datasets, training on a standard central processing unit (CPU) is fine.

This tutorial is performed on a computer containing an Nvidia 1080ti GPU, dual-xeon E5-2670 Intel CPUs, and 64 gb RAM. However, you could perform this experiment on a typical laptop using the CPU only.

## Dataset Preparation

A common machine learning classification problem is to differentiate between two categories (e.g., abdominal and chest radiographs). Typically, one would use a larger sample of cases for a machine learning task, but for this tutorial, our dataset consists of 75 images, split roughly in half, with 37 of the abdomen and 38 of the chest. The data is derived from OpenI, a searchable online repository of medical images from published PubMed Central articles, hosted by the National Institutes of Health (https://openi.nlm.nih.gov). For your convenience, we hosted the images on the following SIIM Github repository: https://github.com/ImagingInformatics/machine-learning. These images are in PNG (Portable Network Graphics) format and ready to be utilized by any machine learning framework. For handling Digital Imaging and Communications in Medicine (DICOM) images, a Python library such as PyDicom (http://pydicom.readthedocs.io/en/stable/index.html) may be used to import the images and convert them into a numpy array for use within the Tensorflow framework. With other frameworks such as Caffe, it may be easier to convert the DICOM files to either PNG or Joint Photographic Experts Group (JPEG) format prior to use.

First, randomly divide your images into training and validation. In this example, we put 65 cases into training and 10 into validation. More information regarding principles of splitting and evaluating your model, including more robust methodologies such as cross-validation, are referenced here [8, 9].

**Fig. 1:**
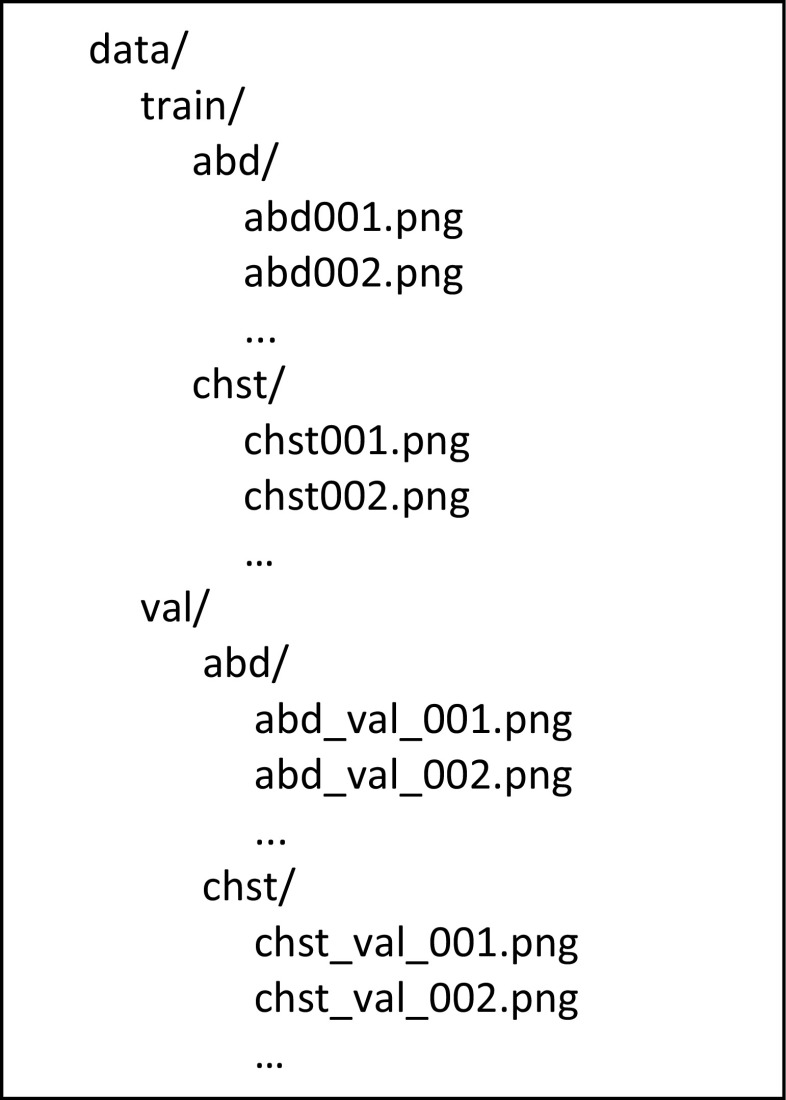
Directory structure for the data

Then, place the images into the directory structure as shown in Fig. 1.

## Setting Up Your Environment

For this example, we will assume you are running this on your laptop or workstation. You will need a computer running Tensorflow, Keras, and Jupyter Notebook (http://jupyter.org/), an open-source web application that permits creation and sharing of documents with text and live code [10]. To make things easier, there is a convenient SIIM docker that has Tensorflow, Keras, and Jupyterlab already installed available at https://github.com/ImagingInformatics/machine-learning/tree/master/docker-keras-tensorflow-python3-jupyter.

**Fig. 2:**
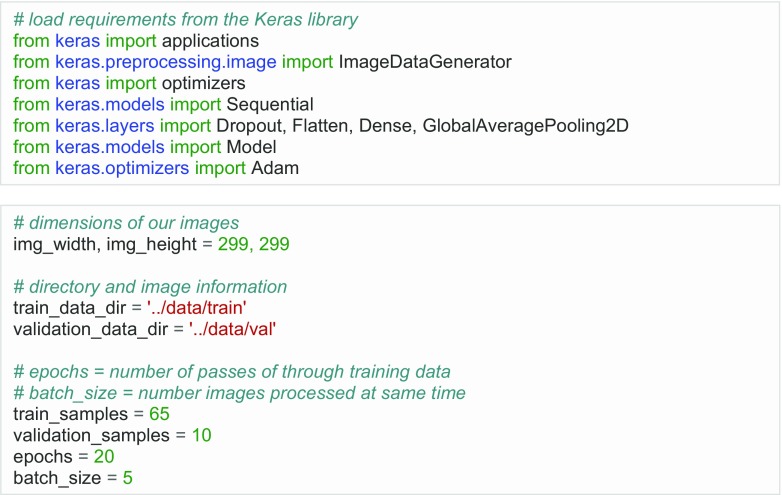
Jupyter Notebook showing initial steps

First, launch a Jupyter Notebook, text editor or Python-supported development environment of your choosing. With Jupyter, the notebooks are organized into cells, whereby each cell may be run independently. In the notebook, load requirements from the Keras library (Fig. 2). Then, specify information regarding the images. Last, define the number of epochs (number of passes through the training data), and the batch size (number of images processed at the same time).

## Build the Model

**Fig. 3:**
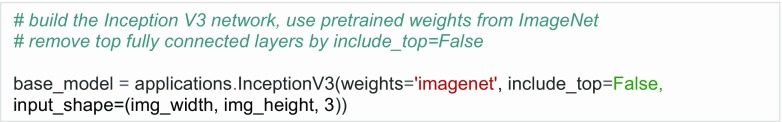
Start with the original Inception V3 model. Then, remove top or fully connected layers from the original network. Use pretrained weights from ImageNet

**Fig. 4:**
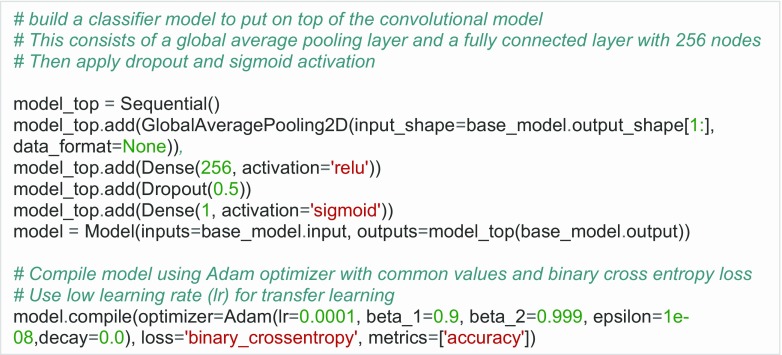
Add new layers on top of the original model. There are many possibilities, but here, we add a global average pooling layer, a fully connected layer with 256 nodes, dropout, and sigmoid activation. We also define an optimizer; in this case, it is the Adam optimizer with default settings

Then, build the pretrained Inception V3 network [11], a popular CNN that achieved a top 5 accuracy of greater than 94% on the ILSVRC. In Keras, the network can be built in one line of code (Fig. 3). Since there are two possible categories (abdominal or chest radiograph), we compile the model using binary cross-entropy loss (Fig. 4), and measure of model performance with a probability between 0 and 1. For classification tasks with greater than 2 classes (e.g., ImageNet has 1000 classes), categorical cross-entropy is typically used as the loss function; for tasks with 2 classes, binary cross-entropy is used.

There are many available gradient descent optimization algorithms, which minimize a particular objective function [12]. In the example, we use the Adam [13] optimizer with commonly used settings (Fig. 4).

## More About Transfer Learning

In machine learning, transfer learning refers to application of a process suited for one specific task to a different problem [14]. For example, a machine learning algorithm trained to recognize every day color images, such as animals, could be used to classify radiographs. The idea is that all images share similar features such as edges and blobs, which aids transfer learning. In addition, deep neural networks often require large datasets (in the millions) to properly train. As such, starting with weights from pretrained networks will often perform better than random initialization if using small datasets [14–16]. In medical imaging classification tasks, this is often the case, as it may be difficult to annotate a large dataset to train from scratch.

There are many strategies for transfer learning, which include freezing layers and training on later layers, and using a low learning rate. Some of this optimization is frequently done by trial and error, so you may have to experiment with different options. For this tutorial, we remove the final (top) fully connected layers of the pretrained Inception V3 model that was intended for a 1000-class problem in ImageNet, and insert a few additional layers with random initialization (Fig. 4), so they can learn from the new medical data provided. We then fine-tune the entire model using a very low learning rate (0.0001), as not to rapidly perturb the weights that are already relatively well optimized.

## Image Preprocessing and Augmentation

**Fig. 5:**
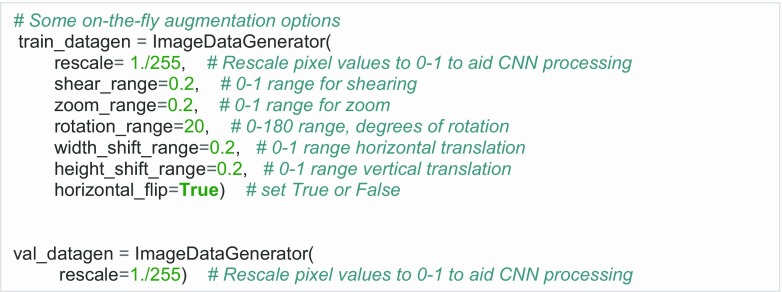
Rescale images and specify augmentation methods

We then preprocess and specify augmentation options (Fig. 5), which include transformations and other variations to the image, which can help preempt overfitting or “memorization” of training data, and have shown to increase accuracy and generalization of CNNs [17]. While augmentation can be done in advance, Keras has an image data generator, which can perform “on-the-fly” augmentation, such as rotations, translation, zoom, shearing, and flipping, just before they are fed to the network.

**Fig. 6:**
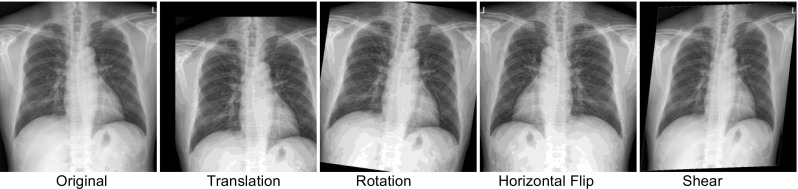
Augmentation examples using the Keras generator

Some examples of transformed images are presented on Fig. 6.

**Fig. 7:**
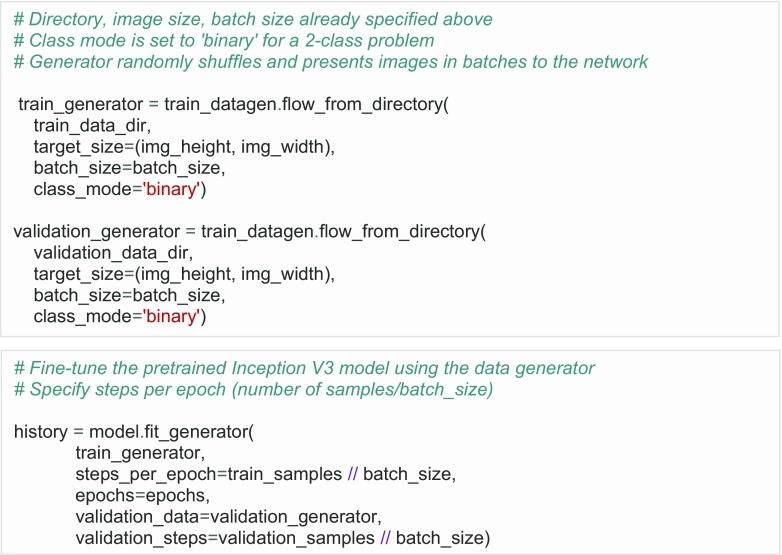
Defining the training and validation generator and fitting the model

Then, more instructions are provided to the generator, such as training directory containing the files, size of images, and batch size (Fig. 7). Then, we fit the model into the generator, which is the last set of code to run the model (Fig. 7).

## Training the Model

**Fig. 8:**
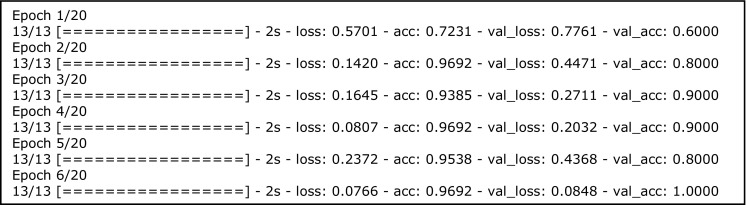
Training metrics. Loss, training loss; acc, training accuracy; val_loss, validation loss; val_acc, validation accuracy. 13 refers to the number of batches (13 batches × 5 images per batch = 65 training images). 20 refers to number of epochs

After executing the code in Fig. 7, the model begins to train (Fig. 8). In only five epochs, the training accuracy equals 89% and validation accuracy 100%. The validation accuracy is usually lower than the training accuracy, but in this case, it is higher likely because there are only 10 validation cases. The training and validation loss both decrease, which indicates that the model is “learning.”

**Fig. 9:**
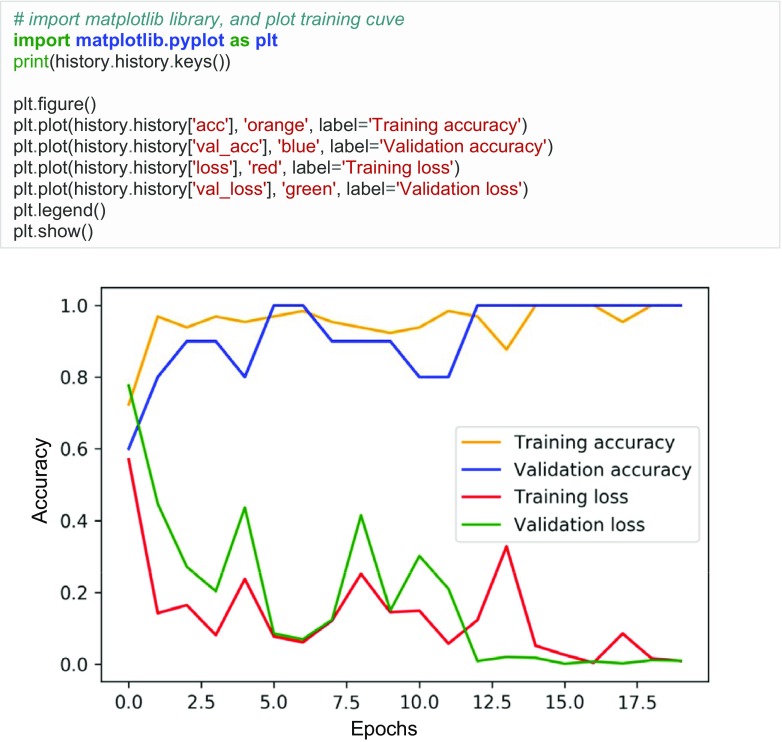
Sample Python code to plot training data. Accuracy increases and loss decreases over time for the training and validation data

The loss and accuracy values are stored in arrays, which can be plotted using Matplotlib (Fig. 9), which is a Python plotting library that produces figures in a variety of formats.

## Evaluating the Trained Model

**Fig. 10:**
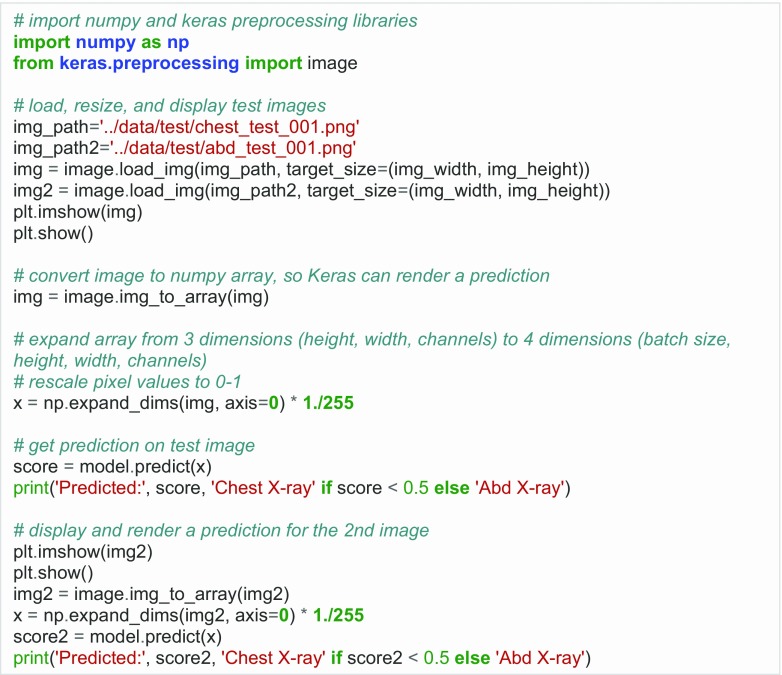
Steps for performing inference on test cases, including displaying of image and generating a prediction score

**Fig. 11:**
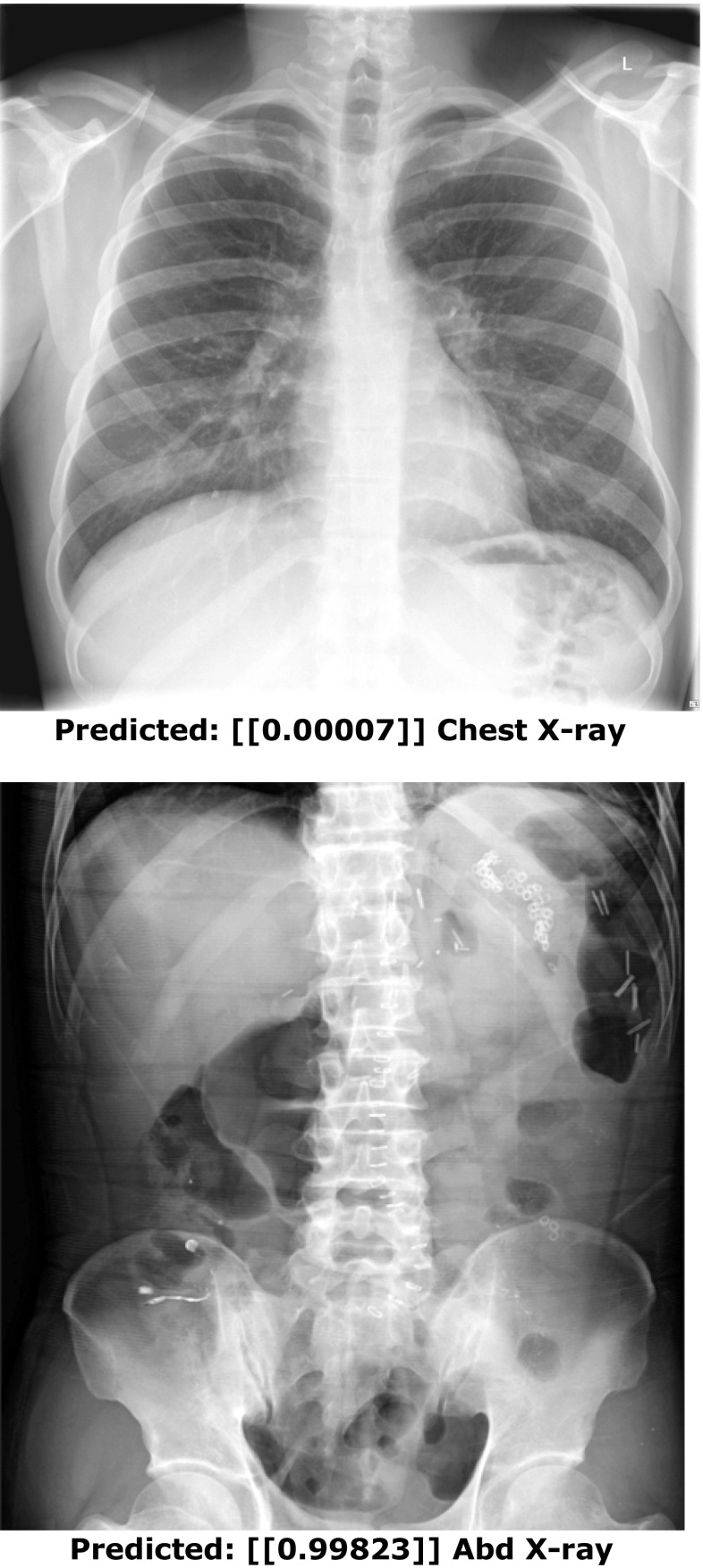
Inference on two test cases. The numbers within the brackets represent the probability of a chest vs. abdominal radiograph (range 0–1). A score close to 0 indicates a high confidence of a chest radiograph, and a score close to 1 indicates a high confidence of an abdominal radiograph

In addition to inspecting training and validation data, it is common to evaluate the performance of the trained model on additional held-out test cases for a better sense of generalization. In Keras, one could use the data generator on a batch of test cases, use a for-loop on an entire directory of cases, or evaluate one case at a time. In this example, we simply do inference on two cases and return their predictions (Figs. 10 and 11). The outputs from such could also be used to generate a receiver operating characteristic (ROC) curve using Scikit learn, a popular machine learning library in Python, or separate statistical program.

## Conclusion

With only 65 training cases, the power of transfer learning and deep neural networks, we built an accurate classifier that can differentiate chest vs. abdominal radiographs with a small amount of code. The availability of frameworks and high-level libraries has made machine learning more accessible in medical imaging. We hope that this tutorial provides a foundation for those interested in starting with machine learning informatics projects in medical imaging.

### Data Availability

The Jupyter Ipython Notebook containing the code to run this tutorial is available on the public SIIM Github repositiory: https://github.com/ImagingInformatics/machine-learning, under “HelloWorldDeepLearning.” A live interactive demo to the model is available at https://public.md.ai/hub/models/public.
